# Editorial: Obesity and metabolism in endocrine-related cancers

**DOI:** 10.3389/fendo.2025.1661893

**Published:** 2025-07-24

**Authors:** Chiara Calabrese, Natalia Simona Pellegata, Sofia Dellavalle, Chiara Liverani

**Affiliations:** ^1^ Preclinic and Osteoncology Unit, Biosciences Laboratory, Istituto di Ricovero e Cura a Carattere Scientifico (IRCCS) Istituto Romagnolo per lo Studio dei Tumori (IRST) “Dino Amadori”, Meldola, Italy; ^2^ Department of Biology and Biotechnology “L. Spallanzani”, University of Pavia, Pavia, Italy

**Keywords:** endocrine-related cancers, metabolism, obesity, adipose tissue, metabolic markers

Obesity and altered metabolism have emerged as significant contributors to the development of endocrine-related cancers, driven by the complex crosstalk between adipose tissue and tumor cells (1, 2). As global obesity rates continue to rise, there is increasing interest in understanding how lipid metabolism and the tumor microenvironment influence cancer progression and response to therapy (3–5). This Research Topic brings together five studies that examine how metabolic factors, such as diabetes, obesity, and lipid metabolism affect the risk, onset, and progression of various endocrine-related cancers. Additionally, these studies explore potential biomarkers and predictive models aimed at enhancing cancer risk assessment and informing treatment strategies.

The study by Shouman et al. highlights the growing impact of diabetes and broader social, biological, and behavioral determinants of health on liver cancer risk. By integrating clinical and demographic variables, the authors highlight how metabolic dysfunction intersects with lifestyle factors to influence liver tumor development, pointing to the urgent need for comprehensive prevention strategies.

In the context of endometrial cancer, a cross-sectional study by Wang et al. investigates the interplay between the triglyceride-glucose (TyG) index as a surrogate measure of insulin resistance, and body mass index (BMI) as an indicator of obesity. The findings reveal that BMI may act as a key mediator in the relationship between metabolic dysfunction and cancer risk, offering new insights into how lipid-glucose homeostasis could serve as an early marker for cancer susceptibility.

Feng et al. contribute to this Research Topic by leveraging machine learning techniques to uncover ferroptosis-related biomarkers in adamantinomatous craniopharyngioma (ACP). Ferroptosis, a form of regulated cell death driven by iron-dependent lipid peroxidation, represents a promising yet underexplored avenue in cancer therapy. By focusing on this lipid-centered mechanism, the study deepens our understanding of the metabolic vulnerabilities of these tumors and highlights potential therapeutic targets that could be exploited to induce ferroptotic cell death and diagnose ACP.

The intricate role of adipokines in tumor progression is echoed in a review conducted by Lagarde et al. on leptin-driven angiogenesis in breast cancer. This work demonstrates how obesity-associated elevations in leptin can fuel vascularization within the tumor microenvironment, reinforcing the concept that adipose-derived signals are central players in breast cancer biology. This finding underscores the potential of leptin signaling inhibitors with anti-VEGF therapy, which may offer improved outcomes for breast cancer patients, particularly those with obesity, for whom conventional anti-VEGF combinations are often less effective.

Finally, Wang et al. examine how distinct metabolic obesity phenotypes correlate with prostate cancer risk in a population-based study from Xinjiang. Using propensity score matching, the researchers delineate how not all forms of obesity confer the same oncogenic risk in prostate cancer, indeed the risk of PCa was higher in both metabolically-healthy obese and metabolically-unhealthy obese individuals compared to metabolically-healthy non obese individuals. The results reveal the complex role of obesity in prostate cancer etiology, emphasizing the importance of refined, phenotype-driven risk stratification approaches in future studies.

Taken together, the studies featured in this Research Topic offer a comprehensive view of the multifaceted relationship between metabolic dysfunction, obesity, and endocrine-related cancers. Common themes emerge across these contributions: the central role of adipose tissue as both a metabolic and inflammatory driver of tumorigenesis; the importance of molecular and metabolic markers, such as leptin, TyG index, and ferroptosis-associated pathways, in predicting cancer risk and progression, as well as the utility of innovative methodologies, including machine learning and population-based modeling, in refining our understanding of cancer etiology.

Moreover, the diverse endocrine-related cancer types addressed, from liver, breast and prostate cancer to endometrial and rare brain tumors, illustrate how metabolic and obesity-related mechanisms transcend tumor boundaries, influencing a broad spectrum of malignancies. The studies also underscore the need to move beyond simplistic classifications of obesity, recommending instead phenotype-specific, mechanism-based risk stratification models.

Looking ahead, these contributions pave the way for deeper exploration of:

- How metabolic pathways, particularly lipid metabolism and ferroptosis, can be therapeutically targeted across tumor types;
- The bidirectional interactions between adipocytes and cancer cells in shaping the tumor microenvironment;
- The combined inhibition of leptin signaling and VEGF in obese breast cancer patients;
- The integration of behavioral, biological, and social determinants in cancer risk modeling;
- The potential for metabolically-driven precision oncology approaches tailored to individual patient profiles.

In conclusion, this Research Topic highlights a significant development in elucidating the links between metabolic health and cancer biology. By connecting mechanistic insights with clinical relevance, these studies contribute to research that could transform both prevention strategies and therapeutic interventions across endocrine and obesity-associated cancers.
